# Ryder: Epigenome normalization using a two-tier model and internal reference regions

**DOI:** 10.64898/2026.03.15.711886

**Published:** 2026-03-18

**Authors:** Yaqiang Cao, Guangzhe Ge, Keji Zhao

**Affiliations:** 1 Laboratory of Epigenome Biology, Systems Biology Center, Division of Intramural Research, National Heart, Lung, and Blood Institute, National Institutes of Health; Bethesda, 20892, USA.; 2 These authors contributed equally to this work.

## Abstract

**Motivation:**

Sequencing-based epigenomic profiling methods are powerful but suffer from technical variability that complicates cross-sample comparisons and can obscure true biological signals. While existing normalization methods using spike-in controls or computational approaches have been proposed, they often rely on assumptions that may not hold across diverse experimental conditions or require additional data types.

**Results:**

We present Ryder, a flexible and robust Python package for the normalization and differential analysis of epigenomic data. Ryder introduces a normalization strategy that leverages stable internal reference regions, such as invariant CTCF binding sites, to correct for technical artifacts genome-wide. Our results show that it effectively models and adjusts both background noise and signal intensity, ensuring accurate signal alignment across samples. We demonstrate that Ryder performs robust, genome-wide normalization – correcting signals in both peak and background regions – across a range of assays including DNase-seq, CUT&RUN, ATAC-seq, MNase-seq, and ChIP-seq, with or without spike-in controls. By reducing technical noise, we show that Ryder improves the detection of genuine biological changes, such as quantitative reduction of chromatin accessibility at key enhancer elements by depletion of BRG1, a key subunit of the chromatin remodeling BAF complexes.

**Availability and Implementation:**

The Ryder source code and documentation are freely available at: https://github.com/YaqiangCao/ryder .

## Introduction

Sequencing-based epigenomic methods such as ChIP-seq (1, 2), DNase-seq (3, 4), ATAC-seq (5, 6), and MNase-seq (7, 8) have revolutionized our understanding of chromatin structure and gene regulation. Large-scale projects have generated comprehensive maps of DNA-protein interactions, chromatin accessibility, and nucleosome positioning, highlighting the complexity of gene regulatory networks (9–12) and further advocating the application of these methods in addressing fundamental biological questions. Despite these advances, technical variability—stemming from sample quality, library preparation, sequencing depth, and batch effects (13) — remains a significant obstacle, often manifesting as inconsistent signal-to-noise ratios between samples. Without appropriate normalization, these artifacts can obscure biological signals, resulting in misinterpretation.

Both experimental and computational normalization methods aimed at mitigating technical biases have been proposed. Spike-in controls, which incorporate exogenous chromatin or cells, provide a direct reference but depend critically on the assumption of equivalent experimental conditions between spike-in and endogenous materials — an assumption that is difficult to validate and may not hold across diverse conditions or protocols. For instance, a recent study found that even minor, unintentional variations in the amount of spike-in DNA added to each sample led to significant, artifactual differences in read counts, making the spike-in data unusable for normalization (14). Additionally, optimal titration of the spike-in material is critical: too little limits its utility, while too much can consume sequencing capacity (15). Furthermore, a single scaling factor derived from spike-ins provides a global normalization, but it may not fully capture localized or site-specific variations (16). On the computational side, methods such as MAnorm operate on the assumption that shared peaks between ChIP-seq samples reflect regions of invariant bindings and should therefore exhibit comparable signal intensities (17). However, this assumption becomes questionable when there are quantitative differences within these shared peaks, and it may not hold true when global changes are present, such as during cellular aging (18–20). More recent methods, such as S3norm, attempt to account for both sequencing depth and signal-to-noise ratio through iterative modeling (21). However, its effectiveness can depend on accurate segmentation of the genome into distinct signal and background regions. This clear separation may be challenging for broadly spread signals, such as those often observed in H3K27me3 or H3K9me3 ChIP-seq data. Other strategies, such as IGN (22), rely on a set of presumed invariantly expressed genes to anchor normalization. However, identifying such stable loci requires ideally matched RNA-seq data, which is assumed to be free of technical variation—an assumption that may not always hold.

Given these challenges, a flexible normalization framework for epigenomic data, capable of accommodating diverse experimental designs, technical variability, and biological contexts, and allowing for easily testable assumptions, is essential. We developed Ryder, a flexible tool designed for cross-sample normalization and the identification of variable features. Ryder leverages stable internal reference regions—such as invariant CTCF sites or user-defined transcription start sites (TSSs) where a reasonable assumption of stability can be tested—to normalize both background and signal genome-wide, aiming for consistent signal alignment and signal-to-noise ratios. Its default two-tier strategy separates background correction from signal alignment, meanwhile it also supports single-scaling or parameter estimation from spike-in controls. This flexible design enables Ryder to normalize a range of assays—including DNase-seq, CUT&RUN, ATAC-seq, MNase-seq, and ChIP-seq—with or without spike-ins, making it potentially effective for both subtle perturbations and global chromatin shifts.

## Results

Within the Ryder package, normalization and differential feature detection are performed sequentially by paw.py and patrol.py, with BigWig files as primary inputs. Paw.py rescales the target BigWig signals using stable internal reference regions, corrects background noise via distribution-based modeling, and outputs both quality-control metrics and normalized signal tracks. Patrol.py then analyzes these tracks across user-defined regions, applying fold-change or Poisson-based statistics to identify differential features, generate M (log ratio) A (average) plots, and export BED files for downstream interpretation.

**Figure 1.**
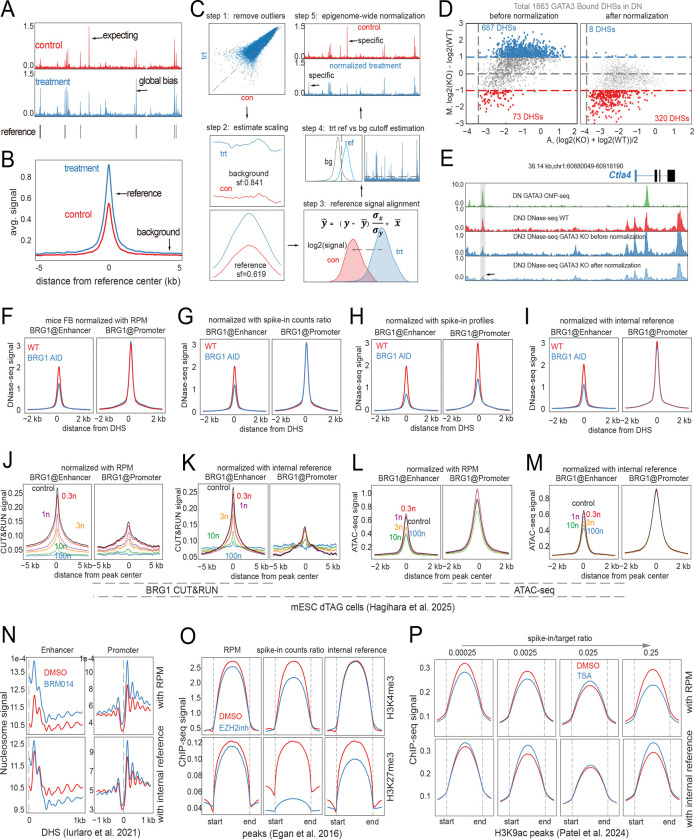
Ryder Normalization Workflow and Applications to Diverse Epigenomic Datasets. (A) Genome browser view of DNase-seq signals in wild-type (control) versus GATA3 knockout (treatment) samples. The tracks represent the average signal from two biological replicates after normalization by library size. Reference peaks shown below the tracks are DNase hypersensitive sites (DHSs) from the control sample that overlap with invariant CTCF binding sites, defined using CTCF ChIP-seq peaks from Cistrome (26) and data from more than 50 cell and tissue types sharing the CTCF sites. (B) Aggregate DNase-seq signals at reference regions (CTCF sites overlapping DNase-seq peaks) before normalization, illustrating the initial discrepancies in background and signal levels between WT and GATA3 KO samples. (C) Schematic of the Ryder normalization workflow:
Identification and removal of outlier internal reference regions using Mahalanobis distance on M-A transformed log_2_ signals.Estimation of scaling factors for background and reference signal regions.Signal alignment via z-score transformation of the target sample to the control sample, or alternatively by fitting a linear model (log_2_ scale) yielding parameters α (slope) and β (intercept).Classification of genomic bins into background or signal based on a cutoff derived from the intersection of log_2_ distributions.Application of normalization across the epigenome using background- and signal-specific transformations. (D) MA-plots of significantly altered GATA3-bound DHSs (fold change ≥ 2) between mouse wild-type and GATA3 knockout double-negative (DN) thymocytes before (left panel) and after normalization (right panel). Normalization reveals additional differential DHSs that were not detected in the unnormalized data due to global differences. (E) Genome browser view of DNase-seq data from DN3 GATA3 KO cells before and after normalization. A highlighted GATA3-bound DHS, undetected prior to normalization, becomes apparent after normalization, demonstrating the method’s ability to uncover biologically relevant changes. (F) Aggregate DNase-seq signals at DHSs in mouse fibroblasts, comparing wild-type and BRG1-AID samples. DHSs were categorized into BRG1-bound enhancers and promoters (within ±2 kb of TSS). Signals were normalized using reads per million (RPM). BRG1 binding sites were identified from ChIC-seq data generated in a previous study (31) and overlapped with DHSs called from wild-type DNase-seq data generated in this study. (G) Same as (F), but signals further scaled by the ratio of spike-in read counts between samples (32). (H) Same as (F), with additional normalization using spike-in signal profiles: human 293T DHSs normalized to derive parameters applied to mouse data. (I) Same as (F), with further normalization using internal reference regions—mouse DHSs overlapping invariant CTCF sites and not overlapping BRG1 peaks. (J) Aggregate BRG1 CUT&RUN profiles in mouse embryonic stem cells (mESCs) following a titration of the dTAG13 degrader, which induces varying levels of BRG1 protein degradation. Signals were RPM-normalized and plotted separately for BRG1-bound enhancers and promoters (as defined in panel F), based on overlap with ATAC-seq data from the same study. Data were obtained from (14), see details in Supplemental Information. (K)Same as (F), but with CUT&RUN signals normalized using Ryder’s internal reference method. The reference set consists of invariant CTCF sites that do not overlap BRG1-bound peaks. (L) Same as (F), but showing the corresponding ATAC-seq profiles from the same dTAG13 degrader titration, illustrating the impact of BRG1 depletion on chromatin accessibility. (M) Same as (L), but with ATAC-seq signals normalized using Ryder’s internal reference method. (N) Aggregate MNase-seq nucleosome occupancy profiles in mESCs treated with DMSO or BRM and BRG1 inhibitor BRM014, separated into enhancers and promoters. Upper panel: RPM-normalized signals. Lower panel: signals further normalized using internal reference regions—DHSs overlapping invariant CTCF sites without BRG1 binding. MNase-seq data were reprocessed from (35), see details in Supplemental Information. (O) Aggregate ChIP-seq signals in human PC9 cells for H3K4me3 (top) and H3K27me3 (bottom), treated with DMSO or EZH2 inhibitor (GSK126). Columns show normalization by: (1) RPM; (2) spike-in read count ratios; (3) internal reference peaks overlapping invariant CTCF sites. Data were obtained from (16), see details in Supplemental Information. (P)Aggregated H3K9ac ChIP-seq signals in HeLa-S3 cells treated with DMSO or Trichostatin A (TSA), which is HDACs inhibitor, with variable yeast spike-in ratios. Upper panel: RPM-normalized signals. Lower panel: normalized using peaks overlapping invariant human CTCF sites. Data were obtained from (15), see details in Supplemental Information.

We assume that constitutive CTCF binding sites—conserved across samples and overlapping with peaks in the dataset—can serve as internal reference regions. This is supported by CTCF’s well-established role as a ubiquitous and stable chromatin architectural protein across diverse cell types and conditions (23–25). Importantly, this assumption is also readily testable, particularly in cases where the perturbed factor is unrelated to CTCF and does not share its DNA-binding motif. Ryder provides human constitutive CTCF binding sites curated from a previous study (23), as well as mouse invariant CTCF sites derived from CTCF ChIP-seq peaks curated in the Cistrome database (26), defined as being consistently shared across more than 50 distinct cell and tissue types. For instance, in our DNase-seq comparison between wild-type and GATA3 knockout (KO) double-negative thymocytes (CD4-CD8-CD25+,DN3), where GATA3 KO halts T cell development at subsequent stages (27), we anticipated changes primarily in chromatin accessibility at GATA3-bound sites. However, we observed a global increase in signal in the KO condition, same as the assumed invariant CTCF binding sites, likely attributable to technical variation (Figure 1A and B) and requiring proper normalization.

For the normalization process, initially, outlier reference sites are identified and removed using the Mahalanobis distance (28, 29) applied to M-A transformed log_2_ signals. Subsequently, signal and background regions (adjacent to the reference regions) are defined, and quality metrics, such as background scaling and signal-to-noise ratio, are assessed. Scaling factors for both background (${sf}_{bg}$) and reference signal regions (${sf}_{sig}$) are then estimated from the aggregated profiles. To estimate signal alignment parameters, the target signals were scaled with $sf_{sig}$ first, then a z-score transformation (on a log_2_ scale) $\hat{y}=(y-\bar{y})\frac{\sigma_{x}}{\sigma_{y}}+\bar{x}$ is performed between target and reference signals, where $\bar{y}$ is the mean value of target signal and $\bar{x}$ is the mean value of reference signal and σ indicates the standard deviation. This yields linear transformation parameters in the log_2_ space: $\alpha =\frac{\sigma_{x}}{\sigma_{y}},\beta =\bar{x}-\frac{\sigma_{x}}{\sigma_{y}}\bar{y}$, and these two parameters can be also obtained by linear fitting from the two distributions. In the final step, genomic regions of the target sample are classified as background or signal based on a noise cutoff derived from the intersection of their log_2_ distributions. Normalization is applied accordingly: background bins are scaled linearly using the background scaling factor, whereas signal bins undergo a two-step process—first, linear scaling using the reference signal scaling factor; second, log_2_ transformation adjusted by α and β, followed by exponentiation. This approach ensures robust correction of global biases and noise between conditions while preserving true biological signal variation (Figure 1C). In total, five parameters are estimated—${sf}_{bg},\;{sf}_{sig},\;\alpha ,\;\beta ,\;noise$ —all of which can be optionally overridden by user input for added flexibility. Application of this normalization procedure revealed that GATA3 knockout resulted in a greater number of GATA3-bound DNase hypersensitive sites (DHSs) with decreased chromatin accessibility (Figure 1D). This effect is exemplified by a distal enhancer bound by GATA3 in the *Ctla4* gene (Figure 1E). *Ctla4* encodes an important immune checkpoint protein (30), and its expression can be decreased by GATA3 KO, further affecting the downstream T cell development.

We further evaluated Ryder’s performance by applying it to DNase-seq experiments comparing wild-type cells to those with acute BRG1 depletion using the auxin-inducible degron (AID) system, incorporating spike-in controls (Figure S1A). This experimental design mirrors that of our previous study (31), which showed that BRG1 plays a critical role in maintaining the accessibility of chromatin at enhancers. Clear DHSs were observed around the *GAPDH* locus in both human spike-in cells and mouse samples (Figure S1B), along with enriched signal profiles (Figure S1C). To assess the effects of BRG1 depletion, we compared DNase-seq signals at BRG1-bound enhancers and promoters (within ±2 kb of TSS) using normalization by reads per million (RPM) first (Figure 1F), then incorporating the ratio of spike-in read counts (32) for additional correction (Figure 1G). Ryder further modeled background and signal-specific scale factors (${sf}_{bg},\;{sf}_{sig}$) and signal alignment parameters (α,β), using either all spike-in DHSs (Figure 1H) or mouse DHSs overlapping invariant CTCF binding sites (Figure 1I). All methods consistently revealed reduced accessibility at BRG1-bound enhancers after BRG1 depletion (Figure S1D&E), but internal reference-based normalization identified more significantly decreased sites at both enhancers and promoters than RPM or spike-in ratio normalizations (Figure S1E).

To further explore this, we re-analyzed a recent dataset that used the dTAG system for progressive degradation of BRG1, followed by CUT&RUN and ATAC-seq (14). Although simple RPM normalization of the CUT&RUN data suggests a dose-dependent decrease in BRG1 binding, this interpretation is confounded by a concurrent artifactual shift in the background signal (Figure 1J). In contrast, Ryder normalization corrects for this background noise and confirms a clear, dose-dependent loss of BRG1 binding specifically at enhancers, with minimal changes at promoters except with extensive degradation of BRG1 (Figure 1K). Strikingly, Ryder also uncovers the functional consequence of this binding loss: a corresponding, dose-dependent decrease in chromatin accessibility at the enhancer regions (Figure 1M). This subtle but significant trend, which was not apparent with simpler methods (Figure 1L), is consistent with our BRG1-AID results and reinforces BRG1’s direct role in maintaining enhancer accessibility. This finding is particularly noteworthy because the original study, after concluding that their own *Drosophila* spike-in data was unusable, relied on a normalization method that could masked this dose-response relationship in strong peaks. By leveraging a stable internal reference, Ryder provides the required sensitivity to detect these quantitative biological trends, highlighting the risks of relying on spike-in controls that may not be suitable for every assay.

Based on the principle that BRG1 primarily affects enhancer accessibility, we further validated Ryder on a published ATAC-seq dataset that used *Drosophila* spike-in controls (33) (Figure S1F, G). As expected, Ryder’s internal reference normalization robustly confirmed a significant reduction in enhancer accessibility, while promoter accessibility remained largely unchanged (Figure S1L). Critically, this stands in stark contrast to standard RPM normalization, which produced a misleading, artifactual increase in promoter accessibility. By avoiding this common artifact (Figure S1I), Ryder provides a more accurate representation of the underlying biology, highlighting the importance of a robust normalization strategy. While spike-in-based methods are available, they can also introduce unwanted bias if not carefully implemented, as shown by the variable results from ratio-based versus profile-based approaches (Figure S1J, K).

Additionally, we demonstrate that Ryder can normalize nucleosome data by applying a background scaling factor ${sf}_{bg}$, estimated from MNase-seq signals at invariant CTCF sites (Figure S1M). Using this normalization approach, we found that inhibition of BRG1 using a chemical inhibitor, BRM014, resulted in increased nucleosome occupancy at enhancer centers and decreased occupancy in flanking regions, consistent with our previous finding that BRG1 acts to maintain a nucleosome-free region at enhancers (31, 34). At promoters, the density of the +1 nucleosome increased by the inhibitor treatment, whereas other nucleosomes near the transcription start site (TSS) showed subtle changes (Figure 1N). Because the nucleosome-to-background signal difference is modest, normalization using only the background scaling factor effectively removes global background variations, yielding results consistent with chromatin accessibility patterns observed by DNase-seq (Figure 1I) and ATAC-seq (Figure 1M and Figure S1L).

We further validated our internal reference normalization method using ChIP-seq datasets in which global changes in chromatin modifications are well-established (15, 16). These scenarios are critical tests for any normalization strategy. First, cells treated with an inhibitor of EZH2, the catalytic subunit of the PRC2 H3K27 methyltransferase, are expected to exhibit a global decrease in H3K27me3 while maintaining stable H3K4me3 levels; Ryder accurately reflected this outcome (Figure 1O). Our method correctly quantified the reduction in H3K27me3 without introducing the common artifact of an apparent increase in the stable H3K4me3 mark, a problem often seen with total-read-based normalizations (Figure 1O, S1N). Second, Ryder confirmed the anticipated global increase in histone H3K9ac levels in cells treated with HDAC inhibitors (Figure 1P). The success in both of these distinct and challenging scenarios demonstrates Ryder’s utility for accurately analyzing ChIP-seq data, particularly when global epigenetic shifts are expected.

## Conclusion and Discussion

Accurate normalization is critical for interpreting epigenomic data, yet many existing methods are often limited by untestable assumptions or inflexibility in the face of global chromatin changes. Here, we present Ryder, a Python tool that overcomes these challenges by using a flexible, two-tier normalization strategy anchored by stable internal reference regions. This approach separately corrects for background noise and signal intensity to achieve consistent signal-to-noise ratios across samples, providing a more robust correction than single-factor methods. Ryder also empowers users to select a single-factor normalization, derived from either signal or background, offering a simpler alternative for specific use cases.

By leveraging stable internal reference regions, such as invariant CTCF sites, Ryder aims to effectively distinguish genuine biological signals from technical artifacts. It supports a variety of epigenomic assays, including DNase-seq, CUT&RUN, ATAC-seq, MNase-seq, and ChIP-seq, with or without spike-in controls. Our validation studies demonstrate Ryder’s ability to accurately detect diverse biological changes, such as reduced enhancer accessibility following BRG1 degradation or inhibition, decreased accessibility at critical GATA3-bound enhancers, and expected chromatin modifications after EZH2 and HDAC inhibitor treatments. We believe Ryder’s flexibility and accuracy can significantly enhance the reliability and interpretability of epigenomic analyses.

While spike-in controls can be useful for normalization, their implementation poses significant challenges regarding selection, titration, and the assumption that they faithfully mimic endogenous chromatin dynamics, which often remains unverified. Our re-analysis of the BRG1-dTAG dataset (14) serves as a critical case study. The original authors themselves concluded their *Drosophila* spike-in was unusable due to inconsistent application. This highlights a fundamental advantage of internal references: they are inherently subjected to the exact same experimental conditions as the regions of interest. Furthermore, normalization strategies using spike-ins also vary, from applying a single global scaling factor to aligning specific spike-in peaks. As our analysis of the BRG1 AID DNase-seq and BRG1 inhibition ATAC-seq data (33) show, spike-in normalization can produce variable and potentially biased results depending on the strategy chosen. In contrast, an internal reference approach provides a more consistent and arguably safer baseline because its core assumption—the stability of specific genomic sites—is transparent and can be readily tested within the dataset itself. Ryder offers what we hope is a practical alternative by utilizing internal reference regions with readily testable assumptions, while still retaining the flexibility to integrate spike-ins when their use is demonstrably beneficial.

In conclusion, Ryder offers a versatile and reliable solution for the normalization of diverse epigenomic data. By effectively distinguishing biological signal from technical noise, it significantly enhances the accuracy and interpretability of epigenomic analyses.

## Supplementary Material

Supplement 1

## Data Availability

The sequencing data of DNase-seq generated in this study have been deposited in the Gene Expression Omnibus database with accession number: GSE300647 (reviewer token: kpmhagsahbyzfgp). Ryder source code and test data used in this study are available at https://github.com/YaqiangCao/ryder.
