# Supplementary material for: Ryder: Epigenome normalization using a two-tier model and internal reference regions
Source: bioRxiv. 2026 Mar 18:2026.03.15.711886. Preprint. [Version 1] doi: 10.64898/2026.03.15.711886 (PMC13015513; doi:10.64898/2026.03.15.711886)
Supplement: Supplement 1 [file media-1.pdf]

Materials and Methods

Mice

The BRG1-AID mice were generated and maintained in our lab as described previously. The *Gata3*$^{fl/fl}$-CreERT mice were kindly provided by Jinfang Zhu in NIAID/NIH. Mice were bred and maintained in the NHLBI animal facilities. All the animal experiments were performed under a protocol approved by the NHLBI Animal Care and Use Committee.

The GATA3 knockout with tamoxifen treatment in DN3 T cells

Tamoxifen was administered to *Gata3*$^{fl/fl}$-CreERT mice by intraperitoneal injection three times (once per day, 2 mg tamoxifen in 150μl corn oil per injection). DN3 T cells were isolated from the thymus 3.5 days later after first tamoxifen injection. To sort DN3 T cells, CD4/CD8 T cells were labeled with anti-CD4/CD8 antibodies and removed using BioMag Goat anti-rat IgG beads from QIAGEN. The remaining cells were stained with linage markers (anti-CD4, CD8a, TCR gamma/delta, CD19, B220, Gr-1, CD11b, CD11c, Nk1.1 and Ter119) and anti-CD3, CD44, CD25. DN3 T cells were sorted as linage^{-}CD3^{-}CD44^{-}CD25^{+}.

The auxin induced BRG1 deletion in mice primary fibroblasts

Primary fibroblasts were isolated and cultured as described previously. In brief, the fibroblasts were isolated from ear in wild type or BRG1-AID mice and then maintained in DMEM medium (high glucose) containing 10% fetal bovine serum. The osTIR1-GFP plasmids were transfected into fibroblasts by Lipofectamine LTX (Cat. No. 15338100) and FuGENE HD (Cat. No. E2311) with the ratio of 1:1. The auxin was added to cells 24 h later after transfection and incubated for 8 ~ 10 h. GFP+ cells were sorted by flow cytometry for further experiments.

DNase-seq

DNase-seq were performed as previously described with minor modifications. Briefly, 10K live cells (mice fibroblasts) were sorted and fixed in 1% formaldehyde. The equal amount

of fixed HEK293T cells were added as spike in control. The fixed cells were permeabilized and the DNase1 (home-made) were added to digest cells at 37 °C for 1 min. Reactions were stopped by adding EDTA, DTT and DNase1 were heat denatured at 55 °C for 10min. After reverse crosslinking, the DNA was purified and DNase-seq libraries were prepared using NEBNext® Ultra™ II DNA Library Prep Kit for Illumina.

Reference genome and public data processing

All bioinformatics analyses were performed using mouse (mm10) and human (hg38) reference genomes annotated by GENCODE (M21 and V30, respectively) (*1*). Reference genomes for Drosophila melanogaster (dm6) and Saccharomyces cerevisiae (sacCer3), used for spike-in normalization, were obtained from the UCSC Genome Browser. Published ChIP-seq, DNase-seq, and ATAC-seq datasets were processed from raw reads into reads-per-million (RPM)-normalized bigWig tracks following established pipelines (*2, 3*). Briefly, sequencing reads were mapped using Bowtie2 (v2.3.5) (*4*), retaining only non-redundant reads with MAPQ ≥ 10 for downstream analyses. Peak calling was performed using the cLoops2 callPeaks module (*5*) with pooled replicates and assay-specific parameters: DNase-seq (-eps 75,150 -minPts 10,20 -sen), ATAC-seq (-eps 75,150 -minPts 20,30 -sen), H3K4me3 ChIP-seq (-eps 150,300 -minPts 20,30 -sen), H3K27me3 ChIP-seq (-eps 300,500 -minPts 20,30 -sen), and H3K9ac ChIP-seq (-eps 75,150 -minPts 30,50 -sen, input as control). Genome-browser-style visualizations were generated using the cLoops2 plotting module (5), and averaged tracks from biological replicates were produced using the bigwigCompare function from deepTools2 (*6*).

Nucleosome center-weighted occupancy score

A revised nucleosome center-weighted occupancy score (*7*) is computed for all nucleosome fragment reads $(147 \pm 15\ bp)$ to quantify aggregated nucleosome density in MNase-seq data. Specifically, a normalized vector of Gaussian weights is generated to weight each nucleosome fragment based on its distance from the fragment center and on whether the fragment length is odd or even. The weight is defined as: $w_d = \begin{cases} e^{-0.5\times(d/20)^2}\ if\ l\equiv 1\ (mod\ 2) \\ 0.5e^{-0.5(d/20)^2}\ if\ l\equiv 0\ (mod\ 2) \end{cases}$, where d is the base distance from the fragment center

and l is the fragment length. The resulting weight vector is normalized by dividing each element by the sum of all weights, ensuring that the total sums to 1. Finally, the genome-wide nucleosome center-weighted occupancy score is normalized by dividing by the total number of nucleosomes, allowing for comparisons between pseudo-bulk samples.

Table S1. Mapping Metrics for DNase-seq Samples Generated in This Study

map to mouse genome	TotalReads	MappingRatio(%)	totalMappedPETs (MAPQ>=10)	uniquePETs	redundancy	tssEnrichmentScore
DNase-seq_DN3_GATA3_WT_rep1	21367057	82.51	15477761	13656214	0.12	3.10
DNase-seq_DN3_GATA3_WT_rep2	25907927	79.1	17998897	15636321	0.13	3.05
DNase-seq_DN3_GATA3_KO_rep1	19260022	82.52	14340692	12294587	0.14	6.32
DNase-seq_DN3_GATA3_KO_rep2	16714976	81.29	12246634	10651694	0.13	6.16
DNase-seq_FB_BRG1_WT_rep1	59804001	17.74	9793516	4065237	0.58	15.39
DNase-seq_FB_BRG1_WT_rep2	80586744	16.36	12183302	3756305	0.69	13.46
DNase-seq_FB_BRG1_AID_rep1	40557941	21.79	8007511	6288068	0.21	15.36
DNase-seq_FB_BRG1_AID_rep2	65310477	20.24	12183822	8743209	0.28	18.24
map to human (spike-in)						
DNase-seq_FB_BRG1_WT_rep1	59804001	28.22	15265034	6741276	0.56	6.51
DNase-seq_FB_BRG1_WT_rep2	80586744	34.25	24937056	8151174	0.67	5.44
DNase-seq_FB_BRG1_AID_rep1	40557941	17.5	6332945	4844088	0.24	9.13
DNase-seq_FB_BRG1_AID_rep2	65310477	16.74	9774468	6826005	0.30	11.86

Table S2. Mapping Metrics for mESC BRG1 dTAG CUT&RUNG and ATAC-seq Samples from (*8*)

CUT&RUNG							
	SampleInformation	TotalReads	Mapping Ratio(%)	totalMappedReads	uniqueReads	redundancy	tssEnrichmentScore
GSM8447862	Control CUT&RUNG rep1	40080542	71.82	47939904	33552797	0.30	2.35
GSM8447863	Control CUT&RUNG rep2	35173549	81.19	49665062	36344419	0.27	1.96
GSM8447864	0.3n dTag CUT&RUNG rep1	34256209	74.9	43323418	30952016	0.29	1.97
GSM8447865	0.3n dTag CUT&RUNG rep2	33071167	75.04	42881668	31076579	0.28	1.84
GSM8447866	1n dTag CUT&RUNG rep1	36274468	78.69	47671180	34005191	0.29	1.80
GSM8447867	1n dTag CUT&RUNG rep2	37401223	75.5	47772276	33483606	0.30	1.74
GSM8447868	3n dTag CUT&RUNG rep1	35439068	82.74	48575338	35402699	0.27	1.38
GSM8447869	3n dTag CUT&RUNG rep2	31777496	76.42	40941186	28812074	0.30	1.40
GSM8447870	10n dTag CUT&RUNG rep1	37021905	81.41	49933356	35397011	0.29	1.13
GSM8447871	10n dTag CUT&RUNG rep2	34743819	79.91	46275358	31385959	0.32	1.04
GSM8447872	100n dTag CUT&RUNG rep1	35586795	88.04	52079120	39105705	0.25	1.03

GSM8447873	100n dTag CUT&RUNG rep2	41295578	79.04	53828950	36112012	0.33	1.03
ATAC-seq							
GSM8447848	WT ATAC rep1	47596196	28.65	24264288	14586184	0.40	8.46
GSM8447849	WT ATAC rep2	32541134	26.11	15059296	9775762	0.35	8.45
GSM8447850	Control ATAC rep1	104414637	29.87	55392112	26424529	0.52	6.74
GSM8447851	Control ATAC rep2	38847627	24.05	16524674	10317716	0.38	8.61
GSM8447852	0.3n dTag ATAC rep1	82653938	23.51	34664040	17327490	0.50	8.74
GSM8447853	0.3n dTag ATAC rep2	85332832	26.99	41107056	20430218	0.50	8.31
GSM8447854	1n dTag ATAC rep1	110718100	24.31	47791140	21409516	0.55	8.00
GSM8447855	1n dTag ATAC rep2	89200833	21.58	33823302	15398627	0.54	10.06
GSM8447856	3n dTag ATAC rep1	111904590	25.8	51308270	23258592	0.55	7.60
GSM8447857	3n dTag ATAC rep2	86297162	24.94	38026776	18324483	0.52	8.12
GSM8447858	10n dTag ATAC rep1	104749972	30.85	57610440	28697349	0.50	5.85
GSM8447859	10n dTag ATAC rep2	92679211	25.55	41835884	20177157	0.52	7.94
GSM8447860	100n dTag ATAC rep1	105471551	29.19	54765438	26688772	0.51	6.24
GSM8447861	100n dTag ATAC rep2	89052066	21.34	33440832	15154868	0.55	10.00

Table S3. Mapping Metrics for MV411 BRG1 Inhibitors Treated ATAC-seq Samples from (*9*)

map to fly (spike-in)	SampleInformation	TotalReads	MappingRatio(%)	totalMappedReads	uniqueReads	redundancy	tssEnrichmentScore
GSM7695921	MV411_ATACseq_1h_AU15330_rep1	17422822	2.91	798838	579152	0.28	2.05
GSM7695922	MV411_ATACseq_1h_AU15330_rep2	17463612	2.85	781684	562673	0.28	1.92
GSM7695923	MV411_ATACseq_1h_AU15330_rep3	17030548	3.22	860174	583944	0.32	1.95
GSM7695924	MV411_ATACseq_1h_BRM014_rep1	17983974	2.86	812070	584247	0.28	2.07
GSM7695925	MV411_ATACseq_1h_BRM014_rep2	17557774	3.32	918844	610007	0.34	1.93
GSM7695926	MV411_ATACseq_1h_BRM014_rep3	19013075	2.73	813036	580961	0.29	1.99
GSM7695927	MV411_ATACseq_1h_DMSO_rep1	11644740	1.88	351320	243360	0.31	1.96
GSM7695928	MV411_ATACseq_1h_DMSO_rep2	15019014	1.86	438878	325268	0.26	1.93
GSM7695929	MV411_ATACseq_1h_DMSO_rep3	15889335	1.68	416380	310511	0.25	1.87
map to human							
GSM7695921	MV411_ATACseq_1h_AU15330_rep1	17422822	90.38	27910554	18754660	0.33	14.51
GSM7695922	MV411_ATACseq_1h_AU15330_rep2	17463612	90.75	28238916	19480882	0.31	14.34
GSM7695923	MV411_ATACseq_1h_AU15330_rep3	17030548	91.99	27851284	18909219	0.32	15.41
GSM7695924	MV411_ATACseq_1h_BRM014_rep1	17983974	90.23	28708372	19500522	0.32	13.68
GSM7695925	MV411_ATACseq_1h_BRM014_rep2	17557774	91.68	28502348	19443913	0.32	14.60
GSM7695926	MV411_ATACseq_1h_BRM014_rep3	19013075	90.48	30731340	22023101	0.28	12.76
GSM7695927	MV411_ATACseq_1h_DMSO_rep1	11644740	94.22	19879902	14074214	0.29	14.43
GSM7695928	MV411_ATACseq_1h_DMSO_rep2	15019014	92.57	25222584	17419360	0.31	13.62
GSM7695929	MV411_ATACseq_1h_DMSO_rep3	15889335	92.84	26847784	18569499	0.31	13.49

Table S4. Mapping Metrics for mESC BRG1 Inhibitor Treated MNase-seq Samples from (*10*)

Samples	SampleInformation	TotalReads	MappingRatio(%)	totalMappedPETs (MAPQ>=10)	uniquePETs	redundancy	fragmentLengthMean	fragmentLengthStd	finalUniqueReadsRemoveBlacklist	yield
GSM4798115	MNase_DMSO_1	72539433	96.65	61516526	58481089	0.05	140.27	33.90	57106816	0.79
GSM4798116	MNase_DMSO_2	74014054	96.2	62288156	59168806	0.05	141.59	36.70	57758467	0.78
GSM4798117	MNase_BRM014-10uM_1	57578034	98.45	50623669	48405771	0.04	129.60	23.94	47342861	0.82
GSM4798118	MNase_BRM014-10uM_2	71671377	97.1	61274946	58259451	0.05	134.78	30.46	56910598	0.79

Table S5. Mapping Metrics for Published ChIP-seq Samples from (*11*) and (*12*)

map to fly (spike-in)	SampleInformation	TotalReads	Mapping Ratio(%)	totalMappedReads	uniqueReads	redundancy	tssEnrichmentScore
GSM1890164	PC9_EZH2inh_H3K4me3_Dmspike	26911224	26.17	6060582	1864308	0.69	3.09
GSM1890165	PC9_control_H3K27me3_Dmspike	42232080	7.33	2200997	2071306	0.06	1.51
GSM1890166	PC9_EZH2inh_H3K27me3_Dmspike	37704531	27.56	7510098	6855981	0.09	1.29
GSM1890167	PC9_control_H3K4me3_Dmspike	20602547	23.98	4252353	1219477	0.71	2.97
map to human							
GSM1890164	PC9_EZH2inh_H3K4me3_Dmspike	26911224	71.51	16691327	5495172	0.67	23.82
GSM1890165	PC9_control_H3K27me3_Dmspike	42232080	92.98	32600655	31236761	0.04	1.34
GSM1890166	PC9_EZH2inh_H3K27me3_Dmspike	37704531	72.55	22117562	20429057	0.08	1.51
GSM1890167	PC9_control_H3K4me3_Dmspike	20602547	73.03	13079959	4099482	0.69	21.02
map to yeast (spike-in)							
GSM8439484	HelaS3, mitotic, DMSO, 0.00025x yeast spike-in, H3K9ac	8343480	2.68	169541	162405	0.04	1.09
GSM8439486	HelaS3, mitotic, DMSO, 0.0025x yeast spike-in, H3K9ac	9960556	1.28	81953	76425	0.07	1.18
GSM8439488	HelaS3, mitotic, DMSO, 0.025x yeast spike-in, H3K9ac	11643652	5.4	538061	519590	0.03	1.13
GSM8439490	HelaS3, mitotic, DMSO, 0.25x yeast spike-in, H3K9ac	12300781	24.28	2703046	2463122	0.09	1.11
GSM8439492	HelaS3, mitotic, DMSO, 2.5x yeast spike-in, H3K9ac	19567820	30.16	5339709	4577292	0.14	1.09
GSM8439494	HelaS3, mitotic, TSA, 0.00025x yeast spike-in, H3K9ac	10677723	1.87	135894	127216	0.06	1.14
GSM8439496	HelaS3, mitotic, TSA, 0.0025x yeast spike-in, H3K9ac	17786080	1.14	109972	96464	0.12	1.22

GSM8439498	HelaS3, mitotic, TSA, 0.025x yeast spike-in, H3K9ac	11430666	4.78	447830	431278	0.04	1.09
GSM8439500	HelaS3, mitotic, TSA, 0.25x yeast spike-in, H3K9ac	20713555	14.21	2613789	2455841	0.06	1.11
map to human							
GSM8439484	HelaS3, mitotic, DMSO, 0.00025x yeast spike-in, H3K9ac	8343480	97.08	7342268	7245589	0.01	6.32
GSM8439486	HelaS3, mitotic, DMSO, 0.0025x yeast spike-in, H3K9ac	9960556	97.55	8788099	8644047	0.02	6.19
GSM8439488	HelaS3, mitotic, DMSO, 0.025x yeast spike-in, H3K9ac	11643652	93.01	9720783	9561352	0.02	5.17
GSM8439490	HelaS3, mitotic, DMSO, 0.25x yeast spike-in, H3K9ac	12300781	74.72	8292870	8156357	0.02	6.14
GSM8439492	HelaS3, mitotic, DMSO, 2.5x yeast spike-in, H3K9ac	19567820	69.67	11970438	11728645	0.02	2.31
GSM8439494	HelaS3, mitotic, TSA, 0.00025x yeast spike-in, H3K9ac	10677723	97.96	9461917	9324459	0.01	5.67
GSM8439496	HelaS3, mitotic, TSA, 0.0025x yeast spike-in, H3K9ac	17786080	98.76	15854488	15605344	0.02	5.07
GSM8439498	HelaS3, mitotic, TSA, 0.025x yeast spike-in, H3K9ac	11430666	95.16	9770364	9623468	0.02	5.27
GSM8439500	HelaS3, mitotic, TSA, 0.25x yeast spike-in, H3K9ac	20713555	85.6	15936259	15685187	0.02	4.60

Table S6. Summary of Deposited Data and Used Public Data

Samples	Source	GEO accessions
DNase-seq of mouse DN3 wild-type and GATA3 knockout cells	This manuscript	GSE300647
DNase-seq of mouse fibroblast wild-type and BRG1-AID cells (with human 293T cells as spike-in)	This manuscript	GSE300647
GATA3 ChIP-seq from mouse DN cells	(*13*)	GSM523221
BRG1 ChIC-seq from mouse fibroblast cells	(*14*)	GSM7713390<br>GSM7713391
DNase-seq of mouse embryonic stem cells (mESC)	(*15*)	GSM1014154
CUT&RUNG of mESC BRG1 dTAG cells	(*8*)	GSM8447862<br>GSM8447863<br>GSM8447864<br>GSM8447865<br>GSM8447866<br>GSM8447867<br>GSM8447868<br>GSM8447869<br>GSM8447870<br>GSM8447871<br>GSM8447872<br>GSM8447873

ATAC-seq of mESC BRG1 dTAG cells	(*8*)	GSM8447850<br>GSM8447851<br>GSM8447852<br>GSM8447853<br>GSM8447854<br>GSM8447855<br>GSM8447856<br>GSM8447857<br>GSM8447858<br>GSM8447859<br>GSM8447860<br>GSM8447861
ATAC-seq of human MV411 cells treated with DMSO, AU15330, and BRM014 (1-hour treatment with Drosophila melanogaster S2 cells as spike-in)	(*9*)	GSM7695921<br>GSM7695922<br>GSM7695923<br>GSM7695924<br>GSM7695925<br>GSM7695926<br>GSM7695927<br>GSM7695928<br>GSM7695929
MNase-seq of mouse embryonic stem cells (mESC) treated with DMSO and BRM014	(*10*)	GSM4798115<br>GSM4798116<br>GSM4798117<br>GSM4798118
DNase-seq of mouse embryonic stem cells (mESC)	(*15*)	GSM1014154
H3K4me3 and H3K27me3 ChIP-seq of PC9 cells treated with DMSO and EZH2 inhibitor	(*11*)	GSM1890164<br>GSM1890165<br>GSM1890166<br>GSM1890167
H3K29ac ChIP-seq of HeLa-S3 cells with variable ratios of yeast spike cells	(*12*)	GSM8439483<br>GSM8439484<br>GSM8439485<br>GSM8439486<br>GSM8439487<br>GSM8439488<br>GSM8439489<br>GSM8439490<br>GSM8439493<br>GSM8439494<br>GSM8439495<br>GSM8439496<br>GSM8439497<br>GSM8439498<br>GSM8439499<br>GSM8439500

Figure S1

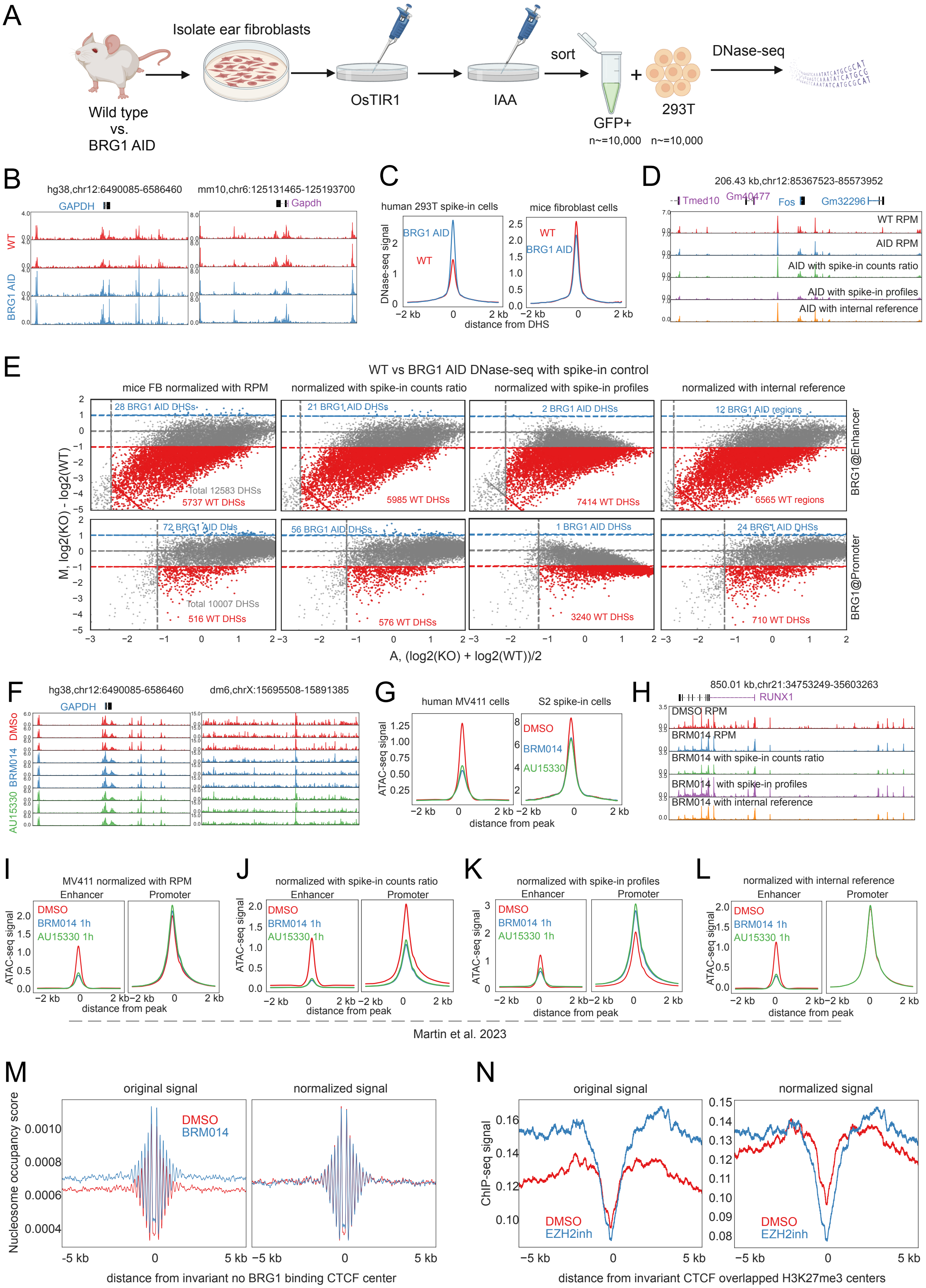
A
Isolate ear fibroblasts
Wild type
vs.
BRG1 AID
OsTIR1
IAA
sort
GFP+
n~=10,000
293T
n~=10,000
DNase-seq
B
hg38,chr12:6490085-6586460
mm10,chr6:125131465-125193700
GAPDH
Gapdh
WT
BRG1 AID
C
human 293T spike-in cells
mice fibroblast cells
DNase-seq signal
BRG1 AID
WT
distance from DHS
D
206.43 kb,chr12:85367523-85573952
Tmed10
Gm40477
Fos
Gm32296
WT RPM
AID RPM
AID with spike-in counts ratio
AID with spike-in profiles
AID with internal reference
E
WT vs BRG1 AID DNase-seq with spike-in control
mice FB normalized with RPM
normalized with spike-in counts ratio
normalized with spike-in profiles
normalized with internal reference
28 BRG1 AID DHSs
21 BRG1 AID DHSs
2 BRG1 AID DHSs
12 BRG1 AID regions
Total 12583 DHSs
5737 WT DHSs
5985 WT DHSs
7414 WT DHSs
6565 WT regions
BRG1@Enhancer
72 BRG1 AID DHs
56 BRG1 AID DHSs
1 BRG1 AID DHSs
24 BRG1 AID DHSs
Total 10007 DHSs
516 WT DHSs
576 WT DHSs
3240 WT DHSs
710 WT DHSs
BRG1@Promoter
M, log2(KO) - log2(WT)
A, (log2(KO) + log2(WT))/2
F
hg38,chr12:6490085-6586460
dm6,chrX:15695508-15891385
GAPDH
DMSo
BRM014
AU15330
G
human MV411 cells
S2 spike-in cells
ATAC-seq signal
DMSO
BRM014
AU15330
distance from peak
H
850.01 kb,chr21:34753249-35603263
RUNX1
DMSO RPM
BRM014 RPM
BRM014 with spike-in counts ratio
BRM014 with spike-in profiles
BRM014 with internal reference
I
MV411 normalized with RPM
Enhancer
Promoter
DMSO
BRM014 1h
AU15330 1h
ATAC-seq signal
distance from peak
J
normalized with spike-in counts ratio
K
normalized with spike-in profiles
L
normalized with internal reference
Martin et al. 2023
M
original signal
normalized signal
Nucleosome occupancy score
DMSO
BRM014
distance from invariant no BRG1 binding CTCF center
N
original signal
normalized signal
ChIP-seq signal
DMSO
EZH2inh
distance from invariant CTCF overlapped H3K27me3 centers

Figure S1. Evaluation of Ryder Normalization Across Diverse Assays and Conditions

(A) Schematic of the spike-in experimental design for DNase-seq. Mouse wild-type (WT) and BRG1-AID fibroblasts were mixed with a constant number of human 293T cells before library preparation.

(B) Genome browser views of representative gene loci showing DNase-seq signals (reads per million) in WT and BRG1-AID samples before normalization.

(C) Aggregate plots of DNase-seq signals centered on all identified DNase Hypersensitive Sites (DHSs) in mouse (left) and human spike-in (right) cells.

(D) Genome browser view comparing the effect of different normalization methods on DNase-seq signal in WT versus BRG1-AID cells. Methods shown are standard RPM, spike-in read-count ratio, Ryder using the spike-in profile, and Ryder using internal reference regions.

(E) MA-plots showing differential BRG1-bound DHSs (enhancers and promoters) between WT and BRG1-AID cells. The comparison illustrates how the number and distribution of significant sites change across different normalization strategies.

(F) Genome browser tracks of ATAC-seq data from human MV411 cells treated with DMSO, BRM/BRG1 inhibitor AU15330, or BRM014, using Drosophila S2 cells as spike-in controls (Data from (*9*)).

(G) Aggregated ATAC-seq signals at identified peaks in Drosophila and human cells prior to normalization.

(H) Genome browser views of ATAC-seq data comparing MV411 cells treated with DMSO or BRM014 under various normalization strategies.

(I) Aggregate ATAC-seq signals on human MV411 cells ATAC-seq peaks, comparing treatment of DMSO, AU15330 and BRM014, which are BRG1 and BRM inhibitors, separated into enhancers and promoters. Signals normalized with RPM. Data from (*9*).

(J) Same as (J), but signals further scaled by the ratio of spike-in read counts between samples.

(K) Same as (J), with additional normalization using spike-in signal profiles: S2 ATAC-seq peaks normalized to derive parameters applied to mouse data.

(L) Same as (J), with further normalization using internal reference regions—MV411 ATAC-seq peaks overlapping human invariant CTCF sites (*16*).

(M) Aggregate plots of MNase-seq signal centered on invariant CTCF sites in mESCs treated with DMSO or a BRG1 inhibitor (BRM014). The comparison shows raw RPM signals (top) versus Ryder-normalized signals (bottom).

(N) Aggregate plots of H3K27me3 ChIP-seq signal centered on invariant CTCF sites in PC9 cells treated with DMSO or an EZH2 inhibitor (GSK126). The comparison highlights the difference between raw RPM signals (top) and Ryder-normalized signals (bottom) in a global-decrease scenario.

References

1. A. Frankish *et al.*, GENCODE reference annotation for the human and mouse genomes. *Nucleic Acids Res* **47**, D766-D773 (2019).
2. Y. Cao, S. Liu, K. Cui, Q. Tang, K. Zhao, Hi-TrAC detects active sub-TADs and reveals internal organizations of super-enhancers. *Nucleic Acids Res*, (2023).
3. K. Cui *et al.*, Restraint of IFN-gamma expression through a distal silencer CNS-28 for tissue homeostasis. *Immunity* **56**, 944-958 e946 (2023).
4. B. Langmead, S. L. Salzberg, Fast gapped-read alignment with Bowtie 2. *Nat Methods* **9**, 357-359 (2012).
5. Y. Cao, S. Liu, G. Ren, Q. Tang, K. Zhao, cLoops2: a full-stack comprehensive analytical tool for chromatin interactions. *Nucleic Acids Res* **50**, 57-71 (2022).
6. F. Ramirez *et al.*, deepTools2: a next generation web server for deep-sequencing data analysis. *Nucleic Acids Res* **44**, W160-165 (2016).
7. K. Brogaard, L. Xi, J.-P. Wang, J. Widom, A map of nucleosome positions in yeast at base-pair resolution. *Nature* **486**, 496-501 (2012).
8. Y. Hagihara, C. Zhang, Y. Zhang, Precise modulation of BRG1 levels reveals features of mSWI/SNF dosage sensitivity. *Nat Genet*, (2025).
9. B. J. E. Martin *et al.*, Global identification of SWI/SNF targets reveals compensation by EP400. *Cell* **186**, 5290-5307 e5226 (2023).
10. M. Iurlaro *et al.*, Mammalian SWI/SNF continuously restores local accessibility to chromatin. *Nat Genet* **53**, 279-287 (2021).
11. B. Egan *et al.*, An Alternative Approach to ChIP-Seq Normalization Enables Detection of Genome-Wide Changes in Histone H3 Lysine 27 Trimethylation upon EZH2 Inhibition. *Plos One* **11**, (2016).
12. L. A. Patel, Y. Cao, E. M. Mendenhall, C. Benner, A. Goren, The Wild West of spike-in normalization. *Nat Biotechnol* **42**, 1343-1349 (2024).
13. G. Wei *et al.*, Genome-wide analyses of transcription factor GATA3-mediated gene regulation in distinct T cell types. *Immunity* **35**, 299-311 (2011).
14. G. Ren *et al.*, Acute depletion of BRG1 reveals its primary function as an activator of transcription. *Nature Communications* **15**, (2024).
15. F. Yue *et al.*, A comparative encyclopedia of DNA elements in the mouse genome. *Nature* **515**, 355-364 (2014).
16. C. Fang *et al.*, Cancer-specific CTCF binding facilitates oncogenic transcriptional dysregulation. *Genome Biol* **21**, 247 (2020).
